# First person – Melody Hancock

**DOI:** 10.1242/dmm.052883

**Published:** 2026-03-31

## Abstract

First Person is a series of interviews with the first authors of a selection of papers published in Disease Models & Mechanisms, helping researchers promote themselves alongside their papers. Melody Hancock is first author on ‘ Multi-omic analyses identify molecular targets of Chd7 that contribute to CHARGE syndrome model phenotypes’, published in DMM. Melody is a PhD candidate in the lab of Kurt Marsden at North Carolina State University, Raleigh, NC, USA, investigating the mechanisms that mediate genetic disease pathogenesis by integrating next-generation sequencing (NGS) datasets to aid therapeutic strategies.


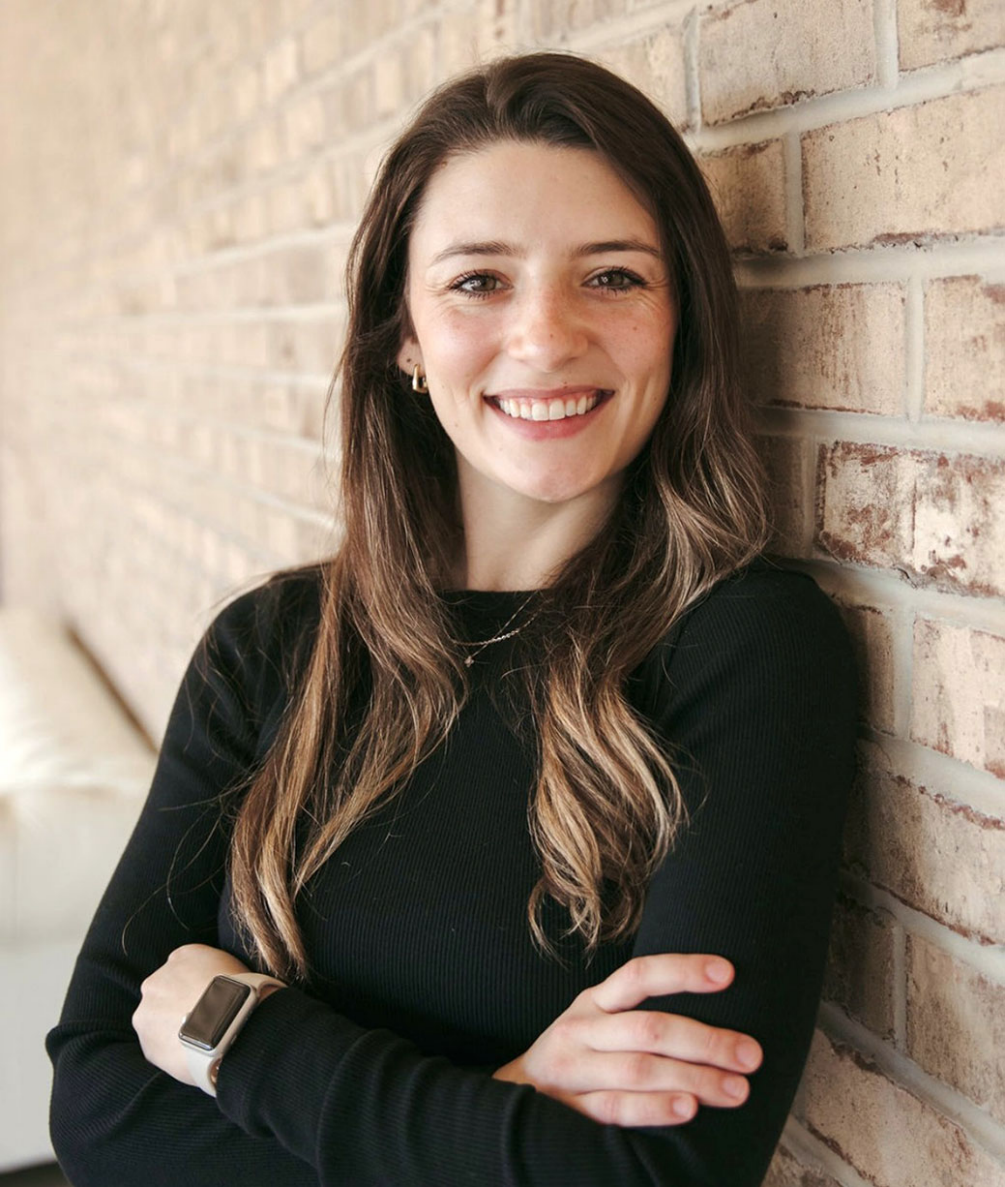


**Melody Hancock**

**Who or what inspired you to become a scientist?**

When I was 7 years old, I had surgery to correct urinary reflux, a condition that is now much more easily diagnosed and treated. At the time, managing it meant many out-of-state specialist visits, daily medication and recurring discomfort. Through that experience, I saw how scientific research is integral to updating treatment and symptom management, as well as a path to preventing the discomfort I experienced for other kids with similar conditions.

**What is the main question or challenge in disease biology you are addressing in this paper? How did you go about investigating your question or challenge?**

In this paper, I identify and validate downstream regulators of the chromatin remodeller *chd7* in a larval zebrafish model; mutations in *CHD7* are a known cause of CHARGE syndrome in humans. CHARGE syndrome is a developmental disorder, and patients often have ear, eye and craniofacial abnormalities, as well as autistic-like behaviours, sensory disorders and anxiety. I identified these regulators of *chd7* by integrating transcriptomic and proteomic data of CHARGE model tissue across two developmental time points. I then induced CRISPR/Cas9 knockdown of candidate genes and found that *capgb*, *nefla* and *rdh5* phenocopy CHARGE syndrome-associated behavioural phenotypes. These genes are likely contributors to CHARGE syndrome model behavioural phenotypes and are potential targets for therapeutic intervention to alleviate specific aspects of CHARGE syndrome.

**How would you explain the main findings of your paper to non-scientific family and friends?**

CHARGE syndrome is a condition that affects how parts of the body develop, including the ears, eyes and facial structures, and many patients also experience behavioural challenges. It is most often caused by changes in a gene called *CHD7*. My research uses zebrafish as a model to understand what goes wrong at the molecular level in this disorder, with the goal of identifying possible new treatment strategies.

**What are the potential implications of these results for disease biology and the possible impact on patients?**

These results identify potential genetic therapeutic targets and provide a reference for integrating multi-omic NGS data to uncover disease mechanisms. Together, they deepen our understanding of disease biology and offer a strong starting point for future studies aimed at developing targeted treatments that could ultimately benefit patients.

[Our results] offer a strong starting point for future studies aimed at developing targeted treatments that could ultimately benefit [CHARGE syndrome] patients

**Figure:**
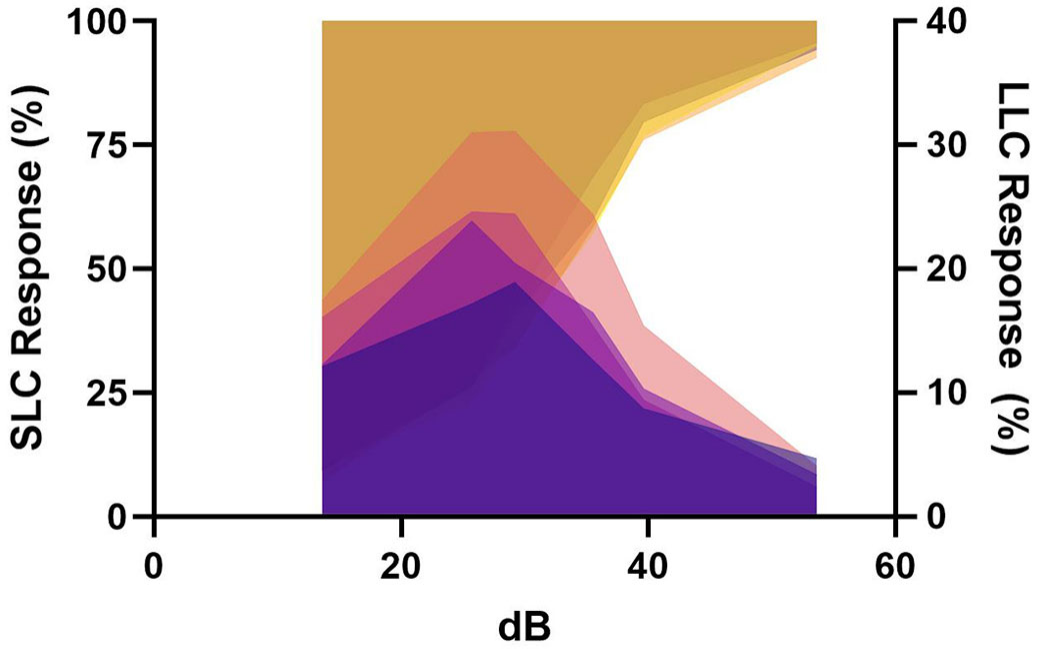
**Short- and long-latency C-bend response rates to acoustic stimuli in larval zebrafish.** Plotted in GraphPad Prism 10. LLC, long-latency C-bend; SLC, short-latency C-bend.

**Why did you choose DMM for your paper?**

Disease models are essential for addressing fundamental questions about disease pathogenesis and underlying molecular mechanisms. Disease Models & Mechanisms is therefore the perfect fit for this work, as well as for my broader scientific interests, which are centred on using model systems to generate insight into human disease.

**Given your current role, what challenges do you face and what changes could improve the professional lives of other scientists in this role?**

As genetics, biology and science, in general, increasingly move toward big data, computation and machine learning/artificial intelligence, computer science and programming courses should be a required part of graduate education core curriculum. This training would significantly improve the professional lives of both wet- and dry-lab scientists by enabling better analysis, interpretation and communication of complex datasets, while also improving reproducibility through more standardized computational workflows.

**What's next for you?**

It is my dream to work at a company that studies rare genetic diseases. At this dream company, we provide genetic diagnostics and gene discovery for patients with suspected inherited conditions. In my dream role, I lead teams of lab technicians and bioinformaticians who generate and analyse genetic data and optimize how these groups work together to improve discovery and diagnosis.

**Tell us something interesting about yourself that wouldn't be on your CV**

I was a division 1 100-metre hurdler during undergrad at Mercer University and broke the school record in 2016.

**What strategies will be most effective in translating large-scale genomic and multi-omics datasets into targeted therapeutic advancements?**

Continuing to expand DNA, RNA and protein databases, especially in non-mammalian model systems, while prioritizing reproducibility and transparent data sharing will strengthen our ability to interpret large-scale datasets and uncover conserved mechanisms that can inform therapeutic development.
